# Evaluating the Dissemination of Mental Health Resources and Service Information in Primary Care: A Quality Improvement Project

**DOI:** 10.1192/bjo.2022.284

**Published:** 2022-06-20

**Authors:** Chloe Challen

**Affiliations:** Imperial College School of Medicine, London, United Kingdom

## Abstract

**Aims:**

*Background:* Demand for mental health support in primary care has increased during the COVID-19 pandemic. Furthermore, in an era of social distancing, the use of digital technology for communication has never been more important. It is therefore vital for mental health services to be easily accessible online, especially because 90% of people with mental health problems are cared for entirely within primary care, despite using <10% of mental health expenditure. *Aims:* 1. To evaluate the dissemination of resources and services to patients during initial mental health consultations. 2. To develop an easy to access and cost-effective resource containing details of both adult and child mental health services.

**Methods:**

An anonymised survey was used to explore the dissemination of mental health resources at the Cotswold Medical Practice. The baseline data collection revealed a lack of easily accessible and shareable information, furthermore, a review of existing literature found that no resource existed containing details of both local and national services. Consequently, two virtual documents were created for adult and child mental health resources. These were added to an accuRx template to allow clinicians to easily send the resources to patients via text message. The resources were then re-evaluated 1–week and 5–weeks post–intervention.

**Results:**

Pre–intervention the average GP provided patients with 2.4 mental health resources and there was no standardisation of the information given. Post-intervention, over 25 resources were provided as both 6–page virtual documents contain a range of resources including: NHS services, local and national charity services, private services, self-help books and mobile apps.

**Conclusion:**

The novel virtual resource produced is a cost-effective resource that helps improve the quality and quantity of information provided to patients about mental health services. The resource produced is compatible with virtual consultations and is sustainable for long term use.

